# Why do women deliver where they had not planned to go? A qualitative study from peri-urban Nairobi Kenya

**DOI:** 10.1186/s12884-019-2695-7

**Published:** 2020-01-13

**Authors:** V. Naanyu, V. Mujumdar, C. Ahearn, M. McConnell, J. Cohen

**Affiliations:** 1 Department of Health Policy and Management, School of Public Health, College of Health Science, Moi University, Eldoret, Kenya; 2 Department of Obstetrics and Gynecology, Thomas Jefferson University Hospital, Philadelphia, PA USA; 3 Department of HIV, ID and Global Medicine, UCSF, San Francisco, California USA; 4 Department of Global Health and Population, Harvard T.H. Chan School of Public Health, Boston, USA

**Keywords:** Delivery facility choice; Access to care, Maternal and child health, Peri-urban Kenya

## Abstract

**Background:**

In urban Kenya, couples face a wide variety of choices for delivery options; however, many women end up delivering in different facilities from those they had intended while pregnant. One potential consequence of this is delivering in facilities that do not meet minimum quality standards and lack the capacity to provide treatment for obstetric and neonatal complications.

**Methods:**

This study investigated why women in peri-urban Nairobi, Kenya deliver in facilities they had not intended to use. We used 60 in-depth audio-recorded interviews in which mothers shared their experiences 2–6 months after delivery. Descriptive statistics were used to summarize socio-demographic characteristics of participants. Qualitative data were analyzed in three steps i) exploration and generation of initial codes; ii) searching for themes by gathering coded data that addressed specific themes; and iii) defining and naming identified themes. Verbatim excerpts from participants were provided to illustrate study findings. The Health Belief Model was used to shed light on individual-level drivers of delivery location choice.

**Results:**

Findings show a confluence of factors that predispose mothers to delivering in unintended facilities. At the individual level, precipitate labor, financial limitations, onset of pain, complications, changes in birth plans, undisclosed birth plans, travel during pregnancy, fear of health facility providers, misconception of onset of labor, wrong estimate of delivery date, and onset of labor at night, contributed to delivery at unplanned locations. On the supply side, the sudden referral to other facilities, poor services, wrong projection of delivery date, and long distance to chosen delivery facility, were factors in changes in delivery location. Lack of transport discouraged delivery at a chosen health facility. Social influences included others’ perspectives on delivery location and lack of aides/escorts.

**Conclusions:**

Results from this study suggest that manifold factors contribute to the occurrence of women delivering in facilities that they had not intended during pregnancy. Future studies should consider whether these changes in delivery location late in pregnancy contribute to late facility arrival and the use of lower quality facilities. Deliberate counseling during antenatal care regarding birth plans is likely to encourage timely arrival at facilities consistent with women’s preferences.

## Background

If women could access a package of effective and affordable interventions for safe childbirth in a timely manner, it is estimated that 80% of the 350,000 maternal deaths, over 45% of the 1.2 million intrapartum-related stillbirths, and up to 72% of the 3.1 million neonatal deaths occurring annually could be avoided [1–3]. Although the proportion of pregnant women who deliver in a facility (rather than home or with a traditional birth attendant) has been increasing rapidly in low-income countries [4], the majority of deliveries occur in very low-quality facilities [5, 6]. While a number of studies have found that pregnant women have strong preferences for facilities that provide high quality technical and inter-personal quality care [7–12], other studies have found that facility distance and cost, as well as factors such as familial influences, and fear of discrimination, influence facility decisions [13, 14].

In the informal settlements of Nairobi, Kenya, the maternal and newborn mortality rate is among the highest in the world, despite most women delivering in a facility [15–17]. Hundreds of public and private maternity facilities of widely-varying quality and cost operate in Nairobi. There is wide variation in quality across these facilities with some providing very high-quality care while others offering sub-standard care [18]. Many pregnant women in Nairobi have limited and inaccurate information about the quality of delivery facility options available [19]. Limited reliable information about delivery facility options during pregnancy can potentially lead to delivery in facilities lacking quality standards and capacity to provide care for emergency obstetric complications [19, 20].

The complex delivery facility landscape in Nairobi contributes to fragmented and often unplanned maternity-care seeking. Normally, women use multiple providers for prenatal care, deliver with a different provider, and choose a delivery facility very late in pregnancy [7]. Many factors have been shown to influence the choice of delivery location in the informal settlements of Nairobi, such as education, wealth, counseling during antenatal care, pregnancy “wantedness,” and parity [21–23]. While several studies have explored the determinants of facility choice in Nairobi and elsewhere [24], there has been very little research on how and why pregnant women switch from a chosen delivery location, and why they often change plans late in pregnancy or during labor.

We conducted a randomized controlled trial in peri-urban Nairobi to explore the impact of cash transfers to pregnant women that incorporated several different incentives designed to improve planning and help women deliver in their desired facility. That study found that more than half of women deliver in a maternity facility they were not even considering in their 8th month of pregnancy and nearly two thirds deliver in a facility that was not where they wanted to deliver. The cash transfers helped women deliver in their desired facility, reduced last minute decisions, and encouraged more timely arrival at higher-quality delivery facilities [18]. This study was conducted as a complementary qualitative investigation to this randomized controlled trial and was intended to investigate why women so often deliver in a facility where they had not planned to go.

## Methods

### Study design and setting

Our qualitative project occurred after the pilot randomized control trial was conducted. In the larger study, 550 pregnant women were recruited in their 5th–7th gestational month and randomized into one of three groups. One group received an unconditional cash transfer given during the 7th month of pregnancy with a label that “this is intended to help you deliver in the facility of your choice.” One group received the same unconditional cash transfer with a label, but was also asked to pre-commit to a delivery facility in their 7th month of pregnancy and was given a conditional cash transfer after delivery if they delivered in a facility of their choice. A third group served as a control. Women were surveyed twice during pregnancy and then once shortly after delivery.

The informal settlements surrounding Nairobi are within 12–15 km of the city center and are primarily made up of low-income residential estates shared with industrial enterprises, especially in locations closer to the city center. These areas are characterized by a large number of public, private, and faith-based health facilities ranging from small pharmacies and outpatient care to large hospitals with maternity wards. These facilities also range widely in cost, size, and services available. Previous research from this trial demonstrated that maternity facilities used by women in the sample varied widely in terms of quality of care, with some facilities well-equipped to handle emergencies and complications for the mother and baby, and others extremely ill-equipped [8].

Using 60 in-depth interviews, the study sought to answer the following question: Why do some women end up delivering where they had not planned to go during pregnancy? We apply the Health Belief Model (HBM) to identify individual level factors that influenced delivery location [25–27]. The HBM postulates six constructs that predict health behavior where people prevent ill-health; 1) If they consider themselves to be susceptible, 2) If they believe the condition may have serious consequences, 3) If they believe that they can do something to reduce their susceptibility, or the severity of the illness, 4) If there may be desirable outcomes, 5) If they recognize barriers that would affect their health action, and 6) If they believe they can successfully complete the action despite barriers. The sixth construct suggests internal and external cues can influence final uptake of a health action [26, 28].

### Study participants

This qualitative component of the study enrolled a sub-sample of 60 randomly selected participants from the original study [8], with 20 women enrolled from each of the three treatment arms to participate in in-depth interviews conducted two to 6 months after their deliveries. We specially targeted women who did not deliver at their intended location and those who were considering multiple delivery facilities. The interviews took place after the original study was completed. Using the list of participants from the main study, women from the three arms were invited by phone and in person to participate. Seventeen women contacted were not engaged in the interviews, 10 because of relocation out of the study area, 5 because they were too busy or not available and 2 because of refusal to participate. These women were replaced with other participants from the same arm.

### Data collection procedures

The lead author, who is proficient in qualitative methodology, trained female research assistants on consent and in-depth interviewing techniques. Research assistants administered one-on-one in-depth interviews lasting approximately one hour at the home of the respondents. An interview guide was used to lay out the open-ended topics in English and Kiswahili. The interview guide used in this study has been attached as an Additional file 1. The interviews covered questions about how women decide where to deliver in an environment with many choices, questions about beliefs, values, and cultural expectations regarding child birth, questions about birth planning, a detailed description of their labor/birth experience, and reasons why those with a birth plan did not deliver where they had initially planned to go. However, this paper focuses only on data on why women deliver where they had not planned to go. Interviewers were trained to hold a “natural dialogue” with the women, allow free flow of the women’s narratives, and probe for relevant topic areas. Each interview was audio-recorded for later transcription and translation to English. Informed consent was signed prior to all study procedures.

### Analysis

After data collection, all transcribed data were read and categorized into meaningful units that were consequently coded using NVivo software. Analysis involved application of both a priori codes (from the question guide) and emergent inductive codes. The study followed Braun and Clarke’s steps of data analysis (2006) which include becoming familiar with data, generating initial codes across the data set and grouping coded data, searching for themes by gathering data that were relevant to each theme, reviewing themes, defining and naming themes identified, and producing an analysis report and selecting appropriate, vivid quotes in support of described themes. Additionally, verbatim quotes were selected and used to illustrate study findings. The Health Belief Model was then used to explain individual level factors that influenced birth location.

## Results

### Characteristics of study participants

A total of 60 participants successfully engaged in the interviews. The characteristics of the sample (measured at the time of baseline survey in the larger randomized trial) were relatively evenly distributed between the arms of the study (Table 1). The total sample had a median age of 26 years, 86.7% were married; 60.0% had a high level of literacy, 60.0% had some level of secondary schooling, and 33.0% were delivering their first child during this study.

**Table 1:** Participant Characteristics (*N* = 60)

Variable	
Age of Respondent, median (IQR)	26 (22–29)
Age of Respondent, mean (range)	26.3 (19–42)
Marital Status, n (%)	
Single	5 (8.3)
Partner	2 (3.3)
Married	52 (86.7)
Separated	1 (1.7)
Literacy, n (%)	
High level of literacy	36 (60.0)
Moderate/low level of literacy	24 (40.0)
Education, n (%)	
Primary school or less	18 (30.0)
Some level of secondary school	36 (60.0)
Post-secondary school	6 (10.0)
Income, median (IQR) in thousands	
Monthly earning (Participant)	0 (0–2)
Monthly earning (Husband)	12 (7.6–24)
Number of facilities considered at baseline	
Mean (SD)	3.5 (1.3)
First Birth, n (%)	
Yes	18 (30)
No	42 (70)

### Why do women go to places they never meant to deliver?

Women and their families take time to think about anticipated deliveries and some make elaborate birth plans. However, many end up delivering in a facility they were not planning to use or in some cases not even considering. Emergent themes were classified into four themes including: 1) individual level factors, 2) health facility factors, 3) influence of significant others, and 4) lack of transport.

The first category includes individual-level factors influencing a woman’s ability to deliver at a chosen location. Women with birth plans that were not well-considered were reported as more likely to deliver in any health facility within easy reach. This was because a woman without a solid decision on delivery location was ready to deliver at any available health facility:

*‘Because she had not made a concrete decision, it is like she is gotten unaware hence she ends up going to any place and delivers there.’* (25045)

Misunderstanding of signs of labor resulted in delivery in unplanned locations. Women were said to ignore contractions that they assumed were false signs of labor. Consequently, some realized too late that they were in active phases of labor. In this way, some women ended up delivering at home.

*‘This is due to the early contractions that may make a woman think they’re false alarms only to find out its labor, so at the end of the day they deliver at home.’* (19006)

Delivery services and associated logistics often require substantial financial resources. For instance, women often must pay transport fares to the health facility, must purchase supplies needed during delivery, and pay for other hospital costs. A woman could choose to deliver at a favorite private health facility only to end up at a public health facility due to financial constraints:

*‘Another thing is money; suppose you had planned to go to a private hospital and you don’t have money? Then you’ll just have to go to a public one.’* (25051)

There were reports about women who did not disclose their birth plans to their friends and relatives. Once in labor, such women relied heavily on whoever was escorting them to a delivery location. They did not fuss about their earlier choices; instead they surrendered decision making on the delivery location to those supporting them:

*‘Maybe she had not told anyone about where she wanted to go and when her time comes, those who are to take her don’t know of her plans so they take her elsewhere.’* (08006)

For women who were unsure about their expected date of delivery (EDD), the labor was unexpected. As they went about their daily business, labor began and they ended up going to the most convenient location within easy reach. Moreover, those who travelled during advanced pregnancy were likely to give birth in unplanned locations especially if they did not know their EDD:

*‘If a woman does not know when she is due, labor comes in suddenly and you will just go to the nearest hospital.’* (27009)

*‘One [reason] is because maybe she had travelled and did not know her days were due.’* (10002)

*‘Sometimes, it could be due to untimely labor which can take place before you reach the intended hospital so you will be forced to go to the convenient hospital.’* (17021)

Women who failed to attend ante-natal care (ANC) shied away from health facility services. It was common for health workers in the maternity unit to require all delivering women to bring their ANC cards in order to facilitate continuity of care from ANC to delivery and onwards to postnatal care. Women who feared health workers due to ANC non-attendance therefore avoided giving birth at their chosen location.

*‘If she had not been attending clinic, she will be afraid to go because she will be asked for her [ANC] book.’* (14071)

Sudden intense pain, confusion, and unexpected complications that occurred during child birth could make a woman change birth location. Those who experienced complications readily decided to change to a new delivery facility where they could get appropriate care. Women described how intense labor pains incapacitated them to a point of confusion about location of birth:

*‘You see during labor; one usually gets confused due to the overwhelming pain so you may end up in a different place.’* (04011)

*‘Confusion due to the labor pains … That pain malfunction’s the brain; it kind of affects her psychologically … You feel like you are going to die, it is painful.’* (01008)

The second category of factors captures health facility influences. Women choose a new delivery location when they observe poor handling of maternity clients by health facility staff. As they seek care and interact with others, they notice disrespectful maternity care at the health facilities. This deters some women who may have planned to deliver at those facilities:

*‘One may get discouraged at how the doctors handle patients and just leave for another hospital.’* (23006)

Health facilities did not provide the same level of maternity services. As such, some women attempted to deliver at their intended facility were referred on to other facilities due to complications that arose in the course of labor. The health care provider wanted to ensure such women got required services accordingly:

*‘They are usually referred during maybe an emergency. You find that the hospital you went to does not have what you need.’* (17017)

*‘Maybe the baby has a problem, maybe the positioning of the baby is not proper, and it will force you to go elsewhere.’* (14066)

On some occasions, health care workers were reported as giving wrong expected dates of delivery. As a result, women went into labor at a time and date that they did not expect. This was disconcerting especially when women made trips to their health facility of choice and were told their EDD was in the near future. As one woman shared, some were sent back home, only to end up delivering near their homes:

*‘On the day of delivery, I went to Kiambu and I was told that I was not yet ready to deliver and when I came back the pain got worse and I had to deliver at a nearby hospital.’* (09003)

Distribution of health facilities was also a concern. Some maternity services were located far away from study communities. Upon onset of labor, a long distance to chosen the chosen delivery facility made it difficult for some women to actually deliver where they had intended. Furthermore, onset of labor at night time meant far off chosen facilities could not be used – it was more convenient to utilize nearby health facilities.

*‘You know it depends, one can go into labor and she will feel that the baby is near and she cannot get to the hospital she had chosen so she will just go to the nearest.’* (21015)

*‘Her husband saw that the only place we can get help is the nearest private because it was at night. It was 2am at night.’* (14081)

A third group of factors captures cues and stimuli from others including husbands, neighbors, and any other escort to the delivery location. Rumors and reports about maternity services could dissuade a mother from fulfilling her initial birth plan. Two quotes are illustrative:

*‘Then there is also that influence from people. You may have decided till the last minute then you change your mind and go to another hospital. People can influence you a lot.’* (04001)

*‘Or maybe you had already prepared yourself to go to a particular hospital then you realize it’s not what you really had planned for from what you have heard, it will force you to change and go somewhere else even if it is far.’* (14066)

As labor commenced, friends and family were reported as raising new or impulsive ideas on where the mother was to deliver. Spouses were especially influential and could change delivery location on the day of delivery:

*‘You may want to go to this hospital but your husband wants you to go to the other one. You know he has also collected information from outside so there will be an agreement. But mostly you will settle on where he decided because likely he is the one who will pay the bills.’* (04001)

*‘At times maybe your husband forces you to go to a hospital, you had not planned for.’* (04003)

*‘Sometimes, whoever is taking you to give birth decides to take you to a different hospital, not the one you had planned to go.’* (05060)

Some women lacked a close family aide to escort them to the maternity service of choice. This made them opt for any nearby convenient facilities that offered delivery services. Some relied on the choices made by neighbors who volunteered to get them to the closest health facility:

*‘Well, in case of early labor and you cannot help yourself, your neighbor may take you to the closest but not of your choice.’* (27001)

*‘Well maybe, the person to take you [to the health facility] may not be there and another one is available. So, you will end up going to the nearest hospital.’* (01066)

Transport was the final factor discussed as influencing access to chosen facilities. Women who lacked readily available means of transport ended up delivering in places they had not chosen. This was a particular barrier for women who had chosen health facilities that were far from their homes. Moreover, some taxi drivers were reported as unwilling to transport women who were in labor to health facilities in order to avoid the possibility of delivery happening in their vehicles while in transit.

*‘Something else you can plan to go to a hospital which is far away but due to lack of means of transport you go to a closer hospital.’* (05030)

*‘Also means of transport. Some taxi drivers refuse to take the women [to health facilities] so you have to go to the nearest.’* (02012)

*‘Maybe the taxi or means of transport are not easily available or the hospitals are quite far.’* (11022)

## Discussion

In this study, a number of factors seem to influence delivery at an unplanned facility. At the individual level, several cognitive factors corresponding to the HBM domains of perceived risk, perceived benefits, perceived barriers, along with the cues to action and self-efficacy, were reported as influencing final birth location.

According to the HBM concept of perceived risk, people prevent ill-health if they consider themselves to be susceptible. Findings suggest women opt for a new delivery location when they sense danger as they observe their body changes, experience sudden intense pain and confusion, or go into abrupt labor. In addition, onset of labor at night time and unexpected birth complications are perceived to be risky and contribute to changes in delivery location. These findings have also been seen in other settings where nighttime delivery and unexpected birth complications have been shown to influence delivery location [29].

Perceived benefits closely align with perceived risks. When women believe that they can do something to reduce their susceptibility of a risky delivery, they make changes to the birth plan. As the EDD nears, women observe their pregnancy and reflect on consequences of giving birth in certain locations of their choice. Consequently, they may change birth location in order to ensure safer delivery in a facility that has skilled staff and the necessary maternity infrastructure for mother and newborn. This has been seen across the world, particularly for those who are choosing between a home or facility-based delivery [30].

Lack of perceived severity with respect to progress in labor applies to themes on misunderstanding signs of labor, precipitate labor, as well as travel close to EDD. If a woman does not perceive her progress in labor and/or potential birth complication risks as far along or severe enough, she may not leave enough time to reach the delivery location of her choice, leading her to be forced to deliver where she had not intended. Similar results have been found in various settings where “quick labor” forced some to change delivery location or to deliver at home instead of a facility [29, 31]. Moreover, limited sharing of birth plan information and knowledge about pregnancy and labor seemed to influence cues to action. Mothers’ lack of communication with birth partners on choice of facility, poor birth plans, indeterminate EDD, and misunderstanding of signs of labor are factors that would influence cues to action.

Self-efficacy applies to a woman’s belief that they she can successfully deliver at a place of choice despite barriers. A lack of firm decision-making and planning during pregnancy leads to women delivering where they had not intended to go. Research has indicated that women’s decision-making power influences delivery location in other settings as well [32, 33]. Strengthening self-efficacy by bolstering the confidence of women to make decisions surrounding their delivery location could reduce the lack of decision-making and help women choose to deliver where they want.

In addition, emotional factors seem to make mothers deliver where they had not intended to go. For instance, fear of health workers who reprimand women aggressively for failing to attend antenatal care makes women avoid health facilities. Past literature reports that disrespect and abuse during childbirth is prevalent in Kenya and barriers to skilled birth delivery include fear of being mistreated or neglected by health workers [20, 34, 35].

The socioeconomic background of the women influenced their ability to deliver at a chosen facility. Financial constraints were a barrier for women who needed money for delivery-preparation, transportation fares, and costs of delivery services. Other studies have shown that hidden costs associated with free maternity care are an obstacle and they often include transportation expenses, costs of food, and incidentals [36, 37].

Social networks were also a factor influencing the final birth location. Husbands, neighbors, and other escorts to the delivery location were reported as over-riding women’s choices about where to deliver. A study conducted in Uganda found that when a woman consulted with her spouse, friend, or family member, she was more likely to deliver with a skilled birth attendant [38] and other studies have shown the importance of male partners in the decision making process of where to deliver [39, 40]. In some cases, women lacked an aide to escort them so they opted for any nearby convenient facilities that offered delivery services. Previous research has shown that this has been found to be particularly problematic during the night time when there is no escort available to bring women to the health facility for delivery [41].

Women face barriers to obtaining care whether they live in rural, urban, or peri-urban settings [42–44]. Usually the issue of distance to maternity facilities is thought to be a problem in rural areas but our study suggests women in urban areas - where distances are not actually far - experience this barrier too. While most women in the informal settlements of Nairobi have a high-quality facility within five kilometers, higher-quality facilities are much more likely to be located outside of the settlements in the city center, requiring women to travel further to reach them [8]. Long distance and lack of means of transport on the day of delivery were prominent environmental barriers to delivery at a chosen location. Distance and travel barriers, including the costs associated with transportation, have been cited frequently as barriers to delivering at health facilities or delivering where women intended. In some settings, the mode of transportation is limited to motorbike or walking, which is not a possibility for some women who are already in labor [13, 29, 45]. Additionally, a recent study found that in an evaluation of 43 Demographic and Health Surveys from different countries in Africa and Asia, transportation plays a major role in where women choose to deliver, which was reflected in this study [46]. If women go to whatever place is closest when their birth plan fails, they are less likely to go to a facility that is good quality. Women living in informal settlements, such as those in our study, are often closest to low quality facilities and delivering in such circumstances is likely to have important implications for outcomes for the mother and baby.

Health system factors influencing delivery choice have been reported in past studies including lack of sufficient staff and training, insufficient referral systems, costs associated with delivery, and distance to health facility [13, 34, 36, 47, 48]. Women are discouraged from delivering where they had planned to go due to poor handling of maternity clients by health facility staff. Research has suggested that poor provider attitude and behavior have been barriers to facility based delivery which may lead to women delivering were they did not intend to go [49]. Findings suggest that health care workers’ use of wrong expected dates of delivery, and their sudden referral of mothers to other facilities, makes some women deliver where they had not planned to go. A recent study conducted in Zambia showed that approximately half of the women who attended an antenatal care visit were made aware of their EDD which enabled them to plan for their delivery. Either having the wrong EDD or not having any information on their EDD can be a major barrier to the planning process for women which may lead to many delivering where they had not intended to go [50].

This study had strengths and limitations. While a number of previous studies have evaluated the reasons why women choose certain delivery facilities, this is one of the first studies to investigate why women may not deliver in places they had hoped and intended to go. We evaluate this question in the context of peri-urban Nairobi, Kenya where the facility used for delivery can have important implications for the quality of care received in delivery and subsequent health outcomes for mother and baby. This research has important implications because of the implications for maternal and child health outcomes when delivery occurs in facilities that cannot meet minimal quality and safety requirements. The sample of 60 recently delivered women allowed for varying viewpoints on the subject matter as they all reflected on why they delivered where they had not intended to go. However, the study also had limitations. We interviewed a random sub-sample of study participants, stratified by the three arms of the intervention. This study would have benefitted from including a purposive sample of women stratified by socio-demographics (e.g. women who were single versus those who were married or first-time mothers versus those who had multiple children) and purposively reaching those who had changed from the intended facility of delivery to another one, and had experienced bad delivery outcomes. More research should also explore how well interventions aimed at individual, health system, and other factors improve women’s ability to deliver in their desired maternity facilities.

## Conclusion

Much of the current literature on maternity care-seeking focuses on the determinants of facility choice without considering the factors that may contribute to women delivering in facilities that differ from their intentions. In this study, we explored factors that seem to influence delivery at a location not intended for among women in peri-urban Nairobi, Kenya. Numerous cognitive, health systems, and other factors influence delivery facility choice in peri-urban Nairobi. This study suggests many reasons for change in birth location are beyond the delivering woman. Significant barriers, including lack of transport at odd hours, lack of support at time of labor and delivery, distance to desired facility, swiftness of labor are all challenging to balance, especially for low income women in informal settlements. It is possible that if nurses spend more time counseling women during ANC regarding the development and implementation of a birth plan, more women would be able to deliver in facilities that are consistent with their preferences and arrive at the facility earlier in labor. Further research should appreciate localized barriers to fulfillment of birth plans and consequently use controlled trials to explore how well diverse interventions reduce delivery at unexpected locations. Accordingly, this study on individual patient choice should be accompanied by a strong health systems approach that includes a strong lens on policy implications.

## Supplementary information

**Additional file 1.** Study interview guide.

## Data Availability

The data used in this study can easily reveal some of the study participants due to the personal narratives used. To avoid breach of confidentiality, data are available from the corresponding author on reasonable request.

## Abbreviations

AMREF: African Medical and Research Foundation
ANC: Ante Natal Care
EDD: Expected Date of Delivery
HBM: Health Belief Model
