# Supplementary material for: Why do women deliver where they had not planned to go? A qualitative study from peri-urban Nairobi Kenya
Source: BMC Pregnancy Childbirth. 2020 Jan 13;20:30. doi: 10.1186/s12884-019-2695-7 (PMC6958584; doi:10.1186/s12884-019-2695-7)
Supplement: Supplementary file 1 — Additional file 1. Study interview guide. [file 12884_2019_2695_MOESM1_ESM.docx]

**Can a Decision-Making Nudge Improve Birth Outcomes? A Pilot Randomized Experiment in Nairobi**

**In-depth Interview Guide for mothers**

Interviewer’s Name: _________________________ Date: __________________

Neighborhood: ______________________________________

Respondent ID: |__| |__| |__| |__| |__|

**Introduction**

Hello. My name is _______________ and I will help guide our discussion today. ______________ is also present today as an observer to help record our conversations. This helps me to capture what you say, not what I think you said. I want to hear about your experiences related to the birth of your baby, your decision-making around where you chose to deliver, and your experience at this facility.

Your opinions and experiences are very important to us. I want to hear what you have to say because it will help us to understand how to do a better job of taking care of pregnant women and their newborn babies, and understand how women make decisions about where they will give birth. You can help us do a better job by giving us your honest opinions.

In this discussion, I consider you the teacher. I want your honest opinions and ideas. I want to be very clear that anything said in this interview will be kept private and confidential. I will not be sharing your answers with anyone, and the recordings of this conversation will not have your names attached to it. You should feel free to say whatever you think and feel. You are welcome to say as much or as little as you want. I am eager to hear your words and to hear what you have to say. If you are uncomfortable with a question, you can decide not to answer it. If this happens, it would be useful to me if you can tell me why you are uncomfortable with a particular question. But if that would also make you feel uncomfortable, then you do not have to say anything. Do you have any questions about what I have said so far?

**Ground rules**

Before I begin, I would like for us to agree on how this interview will proceed. First, if you have a mobile phone, please turn it off or turn it to silent. Next, please speak loudly. I am recording this conversation, and your opinion is very important to me. I want to be accurate in capturing what you say. Finally, understand that there are no right or wrong answers to these questions, only very important information that you can provide to help us improve care for women and babies. May we begin?

A.      Questions about how women decide where to deliver in an environment with so many choices.

- Is it hard to decide where to deliver?
- What facility qualities are most important to you in deciding where to deliver?
- How do women decide where to deliver with so many options?
- Is there anything that would help you decide?
- Why do women go to places they never meant to deliver?

B. Questions about beliefs, values, and cultural expectations regarding child birth

- What is expected of mom during labor or child birth? Are their cultural expectations or traditions regarding how a mother acts or what she does?
- Who is expected to help the mother?
- What happens if cultural practices or rituals are not observed?
- What is the expected role of the father during labor and delivery?
- How is your larger community involved in the birth of a child?

C.      Questions about birth planning

- Describe your birth planning process (How did you decide where to go?  Who did you talk to? What plans did you make to make it possible?)
- Do you feel you had a good plan about your delivery in advance?  Why/Why not?

D.      Detailed description of labor/birth experience

- Describe your labor and delivery experience (when did you decide where to go, how did you get there, when did you get there)?
- Do you have any regrets about where you delivered?
- What did you think this study was about?

Unconditional + Conditional:

- How did you use the money we gave you?
- Did you discuss how to use the money with your partner?  (describe)
- Did the money influence where you decided to deliver?
- Do you have any regrets about the way you used the money?

Conditional:

- How did you decide which facilities to commit to?
- Given what you know now, do you feel you choose the right places?
- How did the incentive influence where you ultimately decided to deliver?
- Where would you have gone to deliver without the incentive?
- Did you discuss the incentive with your partner?  (describe)
- How did you use the money from the incentive?
- Do you have any regrets about how you used the incentive?
- Do you have any regrets about the facility you chose?
- Why do you think the study gave you this incentive?
- Do you think a pre-commitment is helpful for women?

**Closing**

Our goal is to help women deliver babies in a way that is safe for her and her baby. We are interested in how women like you make decisions about where they choose to deliver. We are also interested in what might help women delivery in their chosen facility.

- What are the most important things that need to change to make that possible?
- How do you want child birth to be different for you - or any other mothers – in future?

**I really appreciate the time that you have spent with me today. I appreciate the things that you have shared. You have taught me about child birth practices that are important for you and your communities. Thank you so much for sharing these things with me.**
